# Role of miRNAs in the progression of malignant melanoma

**DOI:** 10.1038/sj.bjc.6605204

**Published:** 2009-07-28

**Authors:** D W Mueller, A K Bosserhoff

**Affiliations:** 1 Institute of Pathology, Molecular Pathology, University of Regensburg, Franz-Josef-Strauss-Allee 11, D-93053 Regensburg, Germany

**Keywords:** miRNA, melanoma, microarray

## Abstract

Analysis of microRNA (miRNA) biogenesis and function is an area of research that started only recently but has subsequently accelerated tremendously. This is because of the impressive impact of miRNA-mediated gene regulation and the obvious potential of those tiny RNA molecules in future diagnostic and therapeutic applications. In this review, recent progress to reveal the role of miRNAs in the tumourigenesis of malignant melanoma, as well as future prospects of melanoma-related miRNA research, will be addressed.

## Malignant melanoma – figures and facts

Malignant melanoma is the most aggressive form of skin cancer and according to the World Health Organization (WHO), the number of melanoma cases worldwide is increasing faster than that of any other type of cancer (Parkin *et al*, 2001). Recent estimates suggest a doubling of melanoma incidence every 10–20 years (Garbe and Leiter, 2009). Melanoma accounts for only about 4% of skin cancer cases but for as many as 74% of all skin cancer deaths. In 2002, the WHO estimated 160 000 new cases of malignant melanoma worldwide and reported 41 000 deaths caused by this dreadful disease (Parkin *et al*, 2005).

Although there is a good chance for the recovery of patients suffering from melanoma if the primary lesion is detected very early (more than 90% survival in stage I melanomas), prognosis of 5-year survival for more advanced melanomas drops down across approximately 60% (stage II) of cases to as low as 10% (stage III) or to even complete fatality (stage IV). This reflects the current lack of therapeutic approaches for treating advanced melanoma. Beyond an early surgical removal of the primary tumour after diagnosis, there is currently no promising standard therapy available for the treatment of melanomas in more advanced stages. This fact gets even more aggravating, considering metastasis to distinct organs at a very early event in the progression of this disease.

Traditionally, five distinct steps of melanoma development and progression are distinguished: A dysplastic nevus (2) showing a high level of structural and architectural atypia arises from a common acquired nevus (1). The subsequent radial growth phase (RGP) primary melanoma (3) is the first recognisable malignant stage in which cells do not possess metastatic potential but are already locally invasive. RGP can be followed by VGP (vertical growth phase) primary melanoma lesions (4) in which melanoma cells infiltrate and invade the dermis and show metastatic potential. This process finally results in metastasis to distant organs by an overgrowth of disseminated tumour cells at these sites (5).

Enormous efforts are taken to unravel the molecular mechanisms that lead to these observed changes in cell architecture and cellular processes and the resulting malignant behaviour of transformed melanocytes. One family of molecules involved in the genesis and progression of melanoma cells, the microRNAs (miRNAs), is now attracting a lot of attention.

## MicroRNA – biogenesis and function

MicroRNAs, a class of small non-coding RNAs, were first described in *Caenorhabditis elegans* in the laboratory studies conducted by Victor Ambros and Gary Ruvkun when they discovered lin-4 (Lee *et al*, 1993; Wightman *et al*, 1993).

It took until the year 2000, when the detection of a second miRNA species – let-7 – by Reinhart *et al* (2000) and the finding that the members of this miRNA family were conserved in a large variety of Metazoens from Drosophila to humans (Pasquinelli *et al*, 2000; Slack *et al*, 2000), to fuel miRNA research. Subsequently, it seemed that this class of molecules regulates gene expression on a posttranscriptional level in presumably every multicellular organism.

MicroRNAs are encoded in the human genome as miRNA genes composed of either a single miRNA species (monocistronic) or several different miRNAs (polycistronic). In most cases, miRNA genes are initially transcribed by RNA polymerase II, resulting in capped and polyadenylated primary transcripts, called pri-miRs, and usually several kilobases in length (for a detailed review on miRNA biogenesis see (Filipowicz *et al*, 2008)). While still in the nucleus, an approximately 60–110-nucleotide stem-loop structure containing the mature miRNA sequence is cut out of the pri-miR by the microprocessor complex, which is mainly composed of the RNAse III enzyme Drosha and its co-factor DGCR8 (the human homologue to Drosophila's Pasha protein). The resulting pre-miRNA (pre-miR) is rapidly translocated to the cytoplasm through the Ran-GTP-dependent nuclear export factor, exportin-5. After a second RNAse III enzyme known as Dicer (together with its co-factor TRBP) excised an approximately 18–24-nucleotide double strand from the pre-miR, the resulting RNA-duplex associates with the miRNA-induced silencing complex (miRISC), the core of which is among others built by Argonaute proteins. Only one strand – the mature miRNA – remains stable on the RISC and induces a posttranscriptional silencing of target genes by binding with imperfect complementary to sequences in the 3′UTRs of their transcripts.

Until today, more than 650 miRNAs have been discovered in the human genome (www.microRNA.org; Betel *et al*, 2008), and these are estimated to regulate about 30% of all human transcripts (Lewis *et al*, 2005). miRNAs are involved in the regulation of multiple cellular processes, such as proliferation, apoptosis, cell-cycle regulation, and differentiation. As a consequence, abnormalities in miRNA activity also contribute to the formation and progression of cancer diseases (reviewed in Visone and Croce, 2009; Mirnezami *et al*, 2009).

## Role of miRNAs in melanoma – summary of available data

### A short history about miRNA expression profiling in malignant melanoma

Initial data on miRNA expression in malignant melanoma samples were included in an article published by Lu *et al* (2005). They examined the expression of 217 mammalian miRNAs in as much as 334 samples, most of them tumour tissues from several different types of cancer (detailed presentation available at http://www.broad.mit.edu). In their remarkable study, they were able to show that miRNA expression profiles reflect the developmental lineage and differentiation state of solid tumours. They also found that poorly differentiated tumours can be successfully classified by their miRNA expression profile in contrast to their mRNA profile. Within the set of specimens were three melanoma tissue samples and two melanoma cell lines. As the interest of Lu *et al* was focused on other aspects, unfortunately no normal melanocyte samples were examined. Thus, miRNAs differentially expressed in melanoma cells compared with the normal biological correlate cannot be determined from their data sets.

During the next 2 years, information published about miRNA expression in melanoma cells was further included only in studies analysing large panels of tissues and cell lines derived from several different types of cancer (Zhang *et al*, 2006; Gaur *et al*, 2007). Zhang *et al* (2006) demonstrated in an extensive array CGH setup that a large number of miRNAs are subject to DNA copy number abnormalities in cancer. In the set of 227 human specimens that they examined, 45 primary cultured melanoma cell lines (contributed by Meenhard Herlyn) were included. Zhang *et al* showed that 85.9% of genomic loci harbouring one or more of the 283 examined miRNA genes exhibited DNA copy number alterations in melanoma and that some of these changes were specific to this kind of cancer. They further confirmed a correlation of copy number alterations and the expression of miRNAs located in this region, indicating that copy number alterations of miRNA genes may account partly for miRNA gene deregulation. It is noteworthy to mention that the latter conclusion was drawn from experiments carried out in ovarial cancer samples included in their study. In general, Zhang *et al*, focused on breast and ovarian cancer samples in their panel of tissues and cell lines. Besides, owing to the low resolution of aCGH techniques, the identification of single miRNA species amplified/lost in melanoma cells would have hardly been possible in this experimental setup.

Gaur *et al* (2007) examined the expression of 241 mature miRNA species in the 59 cell lines of the NCI-60 panel of human tumour-derived cell lines, together with 13 corresponding normal tissues. The NCI-60 panel comprises of cell lines derived from melanoma as well as from cancers of the gastrointestinal tract, kidney, ovary, breast, prostate, lung, central nervous system, and from different leukaemia. They identified a set of 15 miRNAs that were expressed significantly differently in the eight melanoma cell lines included and which separated those from other cancer cell lines (4 up- and 11 downregulated miRNAs). Despite confirming that tumours can be classified by their patterns of miRNA expression, Gaur *et al*, did not characterise melanoma-specific miRNAs in depth. In contrast to the cell lines from colon, haematological, and central nervous system, a comparison of miRNA expression profiles of melanoma cell lines with correlated healthy tissue was not carried out. A study complementing the work by Gaur *et al*, on the NCI-60 cell lines and additionally pointing towards a potential role of miRNAs in chemoresistence was carried out by Blower *et al* (2007).

**Figure 1:**
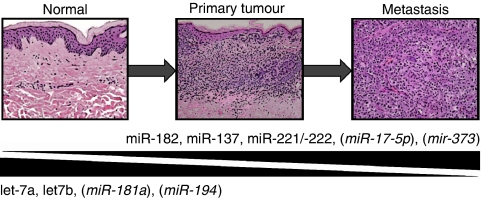
Schematic representation of microRNAs (miRNAs) demonstrated to be up- or downregulated during tumourigenesis of malignant melanoma, as described in the paragraph ‘*Functional characterisation of single miRNA species in melanoma cells*’. miRNAs shown to be deregulated in melanoma progression by a microarray study (Mueller *et al*, 2009) and subsequently confirmed by qRT–PCR as described in the paragraph ‘*A short history about miRNA expression profiling in malignant melanoma*’ are indicated by parenthesis.

In January 2008, a review by Molnar *et al* (2008) was published, in which they summarised data collected on changes in miRNA expression in solid tumours and discussed them with regard to melanoma. They underlined the potential of miRNA profiling to identify miRNAs with a prognostic value in diagnosis and the staging of malignant melanoma, as well as targets for new approaches towards therapy of this disease. The first study carrying out a detailed comparison of the miRnomes of normal human melanocytes to well-characterised melanoma cell lines derived from primary tumours and melanoma metastases was published in February 2009 (Mueller *et al*, 2009). In addition to melanocytes and melanoma cell lines, several model systems used for investigating important steps in melanoma development and progression were included in this work. The experimental setup of this study made it possible to identify miRNAs differentially expressed in each step of melanoma tumourigenesis, such as early development and metastasis. The most important findings can be summarised as follows: (i) expression of a high number of miRNAs is deregulated in melanoma cells compared with normal melanocytes, in which the bulk of miRNAs is upregulated in melanoma cell lines. (ii) The bulk of miRNAs deregulated most strongly was not described to be of importance in tumour development before. (iii) Heterogeneity of melanoma cells causes intrinsic changes in the expression of some miRNAs, which makes it necessary to analyse sets of cell lines/tissue samples to minimise the effects of individual alterations. Quality of data obtained was checked by carrying out a qRT–PCR analysis on some miRNAs deregulated most strongly or indicated to be involved in the progression of other cancer diseases. The expression of those miRNAs was also examined in a set of melanoma tissue samples (most of them being laser microdissected) to ensure that the observed deregulation also harbours relevance *in vivo*. It is interesting to note that several miRNAs, proven to harbour oncogenic or tumour-suppressive potential in other types of tumours, were found to be deregulated in malignant melanoma as well. Thus, these miRNAs may also be relevant in malignant melanoma, although the mechanisms by which they exert their function in this kind of cancer remain to be elucidated. Information about miRNAs confirmed to be deregulated during this study is included in Figure 1, which gives an overview about single miRNA species demonstrated to be deregulated in melanomagenesis.

### Functional characterisation of single miRNA species in melanoma cells

The first report linking the deregulated expression of a single miRNA to its function in melanoma tumourigenesis was published by Bemis *et al* (2008). It is not surprising that the ‘master regulator’ of melanocyte cell growth, maturation, apoptosis, and pigmentation – MITF – was the first gene determined as a target for miRNA-mediated regulation in melanoma (Bemis *et al*, 2008). Bemis *et al*, initially carried out a database search for miRNAs located at the chromosomal region 1p22, a region known to harbour changes related to melanoma susceptibility. MiR-137 was recognised to be encoded in this region, and computational analysis showed MITF as a potential target gene for this miRNA. Bemis *et al*, verified the suppression of MITF as a consequence of direct miR-137 binding and were able to further show that melanoma cell lines expressing MITF show a larger number of a 15-base pair variable number tandem repeat (VNTR) in the 5′ UTR of the miR-137 primary transcript. This amplified VNTR alters the secondary structure of pri-miR-137 so that it cannot be processed further and no mature miRNA can be generated. This observation may explain the highly variable expression of MITF in melanoma cells. Bemis *et al*, already suggested that there might be more miRNAs regulating MITF, and they were right; recently, a paper was published in PNAS that determined miR-182 to be a negative regulator of MITF expression (Segura *et al*, 2009). Starting from a chromosomal aberration (7q31–34, a genomic location frequently amplified in melanoma and also harbouring c-MET and BRAF), Segura *et al*, linked overexpression of miR-182 to increased survival and invasive potential in melanoma cells by repressing MITF and FOXO3 (a transcription factor of the Forkhead family). Overexpression of miR-182 was unable to transform human immortal melanocytes, but strongly stimulated the oncogenic properties of established melanoma cells, especially their ability for anchorage-independent growth and to move through the extracellular matrix. *In vivo* experiments in a mouse model for melanoma lung metastasis further confirmed a clear effect of miR-182 on the ability of melanoma cells to build metastases in distant organs. Considering the hypothesis that MITF has to be upregulated in early melanoma development and subsequently downregulated when the tumour becomes invasive, the interplay between miR-137 and miR-182, and possibly some other miRNAs, may have a key role in the MITF regulating network.

Further, miRNAs with known target genes in melanoma are miR-221 and miR-222, which are clustered on the X chromosome and transcribed as a common precursor. After being reported to be overexpressed in a variety of cancers in which they exert their function by repressing the c-Kit receptor, a group around Alessandra Carè found that both miRs are also directly involved in melanoma pathogenesis (Felicetti *et al*, 2008b; Felicetti *et al*, 2008a). The rationale behind investigating miR-221/-222 was straightforward, as the majority of invasive and metastatic melanomas shows a downregulation of the c-Kit receptor. Felicetti *et al* showed that this diminished expression of c-Kit is a result of the upregulation of miR-221/-222 expression during melanoma progression from primary tumour to a more invasive melanoma phenotype. In addition, repression of p27, another miR-221/-222 target gene identified in prostate carcinoma and glioblastoma studies, could be confirmed in melanoma cells. Overexpression of miR-221/-222 in melanoma cells harbouring a low endogenous level of both miRNAs led to increased proliferation (through acceleration of the cell cycle), increased invasive and chemotactic capabilities, and accelerated tumour growth in a mouse melanoma model. Conversely, treatment of melanoma cells harbouring a high level of miR-221/-222 with antagomiRs against both miRNAs resulted in a reduced proliferation rate and a decrease in invasion and migration abilities. It should be noted that tumour growth was efficiently reduced when antagomiRs against miR-221/-222 were injected directly into the tumour mass. Thus, it was shown that miR-221/-222 regulates two divergent pathways leading to melanoma progression and that treatment with antagomiRs against miR-221/-222 can inhibit melanoma progression *in vitro* as well as *in vivo*. The enhanced expression of miR-221/-222 was shown to be caused by a loss of the transcription factor PLZF in melanoma cells, as the latter binds to the miR-221/-222 promoter and acts as a transcriptional repressor in normal melanocytes. Similar results were obtained by Igoucheva and Alexeev (2009), who interestingly revealed that c-Kit regulation is mainly based on miRNA-dependent mechanisms and is independent of the AP-2 transcription factor.

The first miRNA verified to be actively involved in the formation of cancers was miRNA let-7. Frank Slack's group was able to link the deregulated expression of let-7 in human cancer cells to initial mechanisms of tumour development by the identification of one of its main targets – Ras (Johnson *et al*, 2005). Research on the let-7 family of miRNAs also revealed an important function of its members in melanoma tumourigenesis. The group around Manfred Kunz was able to show that let-7b both directly and indirectly targets cell cycle regulators in melanoma (Schultz *et al*, 2008). Analysing 10 melanocytic nevi and 10 primary melanomas for differentially expressed miRNAs, they revealed five members of the let-7 family to be significantly downregulated in melanoma cells. Further experiments with let-7b mimics showed a reduced expression of cyclins A, D1, D3, and cyclin-dependent kinase (Cdk) 4 in transfected melanoma cells. Schultz *et al* were able to verify a direct interaction of let-7b with the cyclin D1 3′UTR, whereas at least some of the effects on other cyclins are likely to be indirect. Nevertheless, a transfection of let-7b mimics was shown to decelerate cell cycle in melanoma cells, reflected by a significant reduction in the number of melanoma cells in the S-phase and an increase in the number of cells in the G1 phase. The transfection of artificial let-7b molecules into melanoma cells also diminished their ability for anchorage-independent growth. These findings assigned let-7b a role as an important negative regulator of melanoma cell growth and proliferation through inhibition of cell-cycle progression.

Another member of the let-7 family of miRNAs, namely, let-7a, was demonstrated to regulate the expression of integrin beta3 by a direct interaction with a binding site in its 3′UTR (Muller and Bosserhoff, 2008). It was further shown that the loss of let-7a expression is the main regulatory mechanism leading to increased integrin beta3 expression in melanoma cells, whereas promoter-dependent mechanisms have only a minor role. Integrin beta3 is highly related to melanoma progression and leads to enhanced migratory and invasive potential of melanoma cells. In line with these observations, transfection of let-7a mimics into melanoma cells reduced their invasive potential by approximately 75%. Treatment with let-7a anti-miRs, on the other hand, was sufficient to induce migration of otherwise normal melanocytes. These findings revealed that let-7a exerts a strong impact on melanoma tumourigenesis by regulating target genes involved in different cellular mechanisms, contributing to the progression of melanoma cells. On the one hand, expression of the Ras oncogene is regulated by let-7a in melanoma (as well as in other types of cancer). The loss of let-7a thereby leads to enhanced cell proliferation. On the other hand, let-7a is the basic mechanism for the regulation of integrin beta3 expression; thus, migration and invasion of melanoma cells is enhanced by depletion of let-7a.

**Figure 2:**
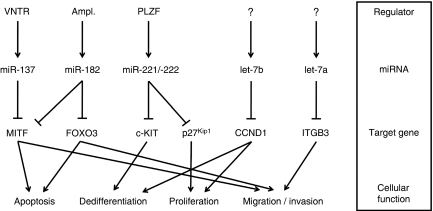
Overview on microRNAs (miRNAs) with validated target genes and their impact on cellular functions in malignant melanoma as summarised in the paragraph ‘*Functional characterisation of single miRNA species in melanoma cells*’. The cellular functions indicated are rather related to miRNA than to single confirmed target genes. Those miRNAs indicated by parenthesis in Figure 1 are not included in this diagram, as their target genes as well as their pathophysiological relevance for melanoma have to still be elucidated in detail (VNTR: variable number tandem repeat; Ampl.: amplification, PLZF: promyelocytic leukaemia zinc finger; MITF: microphthalmia-associated transcription factor; CCND1: cyclin D1; ITGB3: integrin beta 3).

The known miRNA : target interactions in melanoma and their implications in main cellular processes identified in the course of the studies cited in this paragraph are presented in Figure 2.

### Further interesting findings with regard to miRNAs in malignant melanoma

Ozsolak *et al* (2008) identified promoter regions of 175 human miRNAs by combining nucleosome mapping with chromatin signatures for promoters. Besides several findings of utmost importance for general miRNA research, they were able to determine nine miRNAs (e.g., miR-146a, miR-221/222, and miR-363) that are highly likely to be regulated by MITF and seem to be specific to melanoma.

Lodygin *et al* (2008) showed that expression of miR-34a is silenced in a broad range of human tumours because of an aberrant CpG methylation of the corresponding promoter region. It is interesting to note that 43.2% of the melanoma cell lines investigated, as well as 62.5% of primary melanoma samples, showed methylation of the miR-34a promoter, whereas the two samples of normal primary melanocytes included in the study did not show promoter methylation. Although the tumour suppressive function of miR-34a in melanomagenesis has still to be elucidated in detail, Migliore *et al* (2008) demonstrated that a reduced expression of miR-34b, miR-34c, and miR-199a^*^ represents an additional pathway of regulating the expression of the *MET* oncogene in melanocytic cells as well. Ectopic expression of these miRNAs in primary melanoma cells showing a low level of endogenous miR-34b, miR-34c, and miR-199a^*^ led to a decreased MET protein expression and resulted in the impairment of MET-mediated motility in these cells.

Although not discussed in detail in this review because of space limitations, we would like to point out that there are also two interesting publications about miRNAs in uveal melanoma. Worley *et al* (2008) depicted the potential of miRNA expression patterns in predicting the metastatic risk of uveal melanomas, whereas Yan *et al* (2009) were able to demonstrate miR-34a, a tumour suppressor in uveal melanoma cells.

This summary of data shows that, up to now, only very few miRNAs deregulated in the tumourigenesis of malignant melanoma have been analysed in detail and assigned at least some melanoma-relevant target genes. Nevertheless, this limited state of knowledge already points to a pivotal role of miRNAs in the regulation of genes highly involved in the formation and progression of malignant melanoma.

## Future prospects – diagnosis and therapy

Owing to the emerging, serious impact of miRNAs on tumourigenesis, an extensive potential of these small RNA molecules on the development of new diagnostic tools and therapeutic approaches is expected.

With regard to diagnostic purposes, it was shown that poorly differentiated tumours as well can be efficiently classified by their miRNA expression profiles (Lu *et al*, 2005). Expanding those findings, Rosenfeld *et al* (2008) were able to construct a classifier consisting of 48 miRNAs that is capable of determining the origin of metastatic tumours of unknown primary origin with high accuracy. This classifier could prove to be relevant for the diagnosis of unknown primary melanomas that represent about 5% of all melanoma cases. Furthermore, miRNAs were demonstrated to be surprisingly stable in serum and can thus serve as diagnostic markers in blood samples (Mitchell *et al*, 2008; Gilad *et al*, 2008). Unfortunately, no studies dealing with diagnostic applications of miRNAs are available for melanoma. Here, as true for all tumour markers for melanoma, markers distinguishing between nevi and early melanoma, and also miRNAs separating primary melanomas that will lead (or already led) to metastasis from non-metastasising tumours, are urgently needed. It is important to note that formalin-fixed and paraffin-embedded specimens of melanocytic lesions turn out to be suitable starting material for miRNA microarray profiling, as shown by Glud *et al* (2009). This represents a crucial finding, considering the usually poor availability of fresh melanoma tissue samples.

miRNAs are also assigned large therapeutic potential, which is founded in several aspects: (i) the possibility to utilize knowledge acquired during the development of siRNA therapies for miRNA-based approaches. Large progress was achieved in the delivery of small RNA molecules into cells for therapeutic reasons (Whitehead *et al*, 2009; Love *et al*, 2008). Actually, Elmen *et al* (2008) successfully antagonised the expression of the liver-specific miRNA, miR-122, in African green monkeys by a simple systemic delivery of unconjugated, PBS-formulated LNA-antimiRs. (ii) The fact that miRNA antagonising molecules (antagomiRs/antimiRs) and miRNA mimics (miRNA mimetics) can be used. Hence, miRNAs upregulated as well as downregulated in malignant cells could be targeted for therapy. (iii) The ability of miRNAs to regulate several target genes. Thus, by targeting one miRNA, it is likely possible to target multiple pathways involved in the formation and/or progression of the tumour. There are already potential therapeutic targets among the few melanoma-relevant miRNAs analysed in detail: whereas the miR-221/-222 cluster seems to be a promising target in more advanced melanomas, miRNAs of the let-7 family, especially let-7a, could also be of interest in early melanoma stages.

### Pitfalls to overcome for the utilisation of miRNAs in therapy

The last years of research on cancer-related miRNAs showed that this field is complicated by several pitfalls – most importantly in identifying target genes for particular miRNAs to unravel the involvement of the latter in global cellular function. Two major facts that have to be kept in mind are (i) that miRNA-mediated regulation of gene expression can often only be found at the protein level and (ii) that present bioinformatic algorithms are insufficient to reliably predict related target genes. On the one hand, this entails that cDNA array data collected over recent years can only contribute to the identification of miRNA target genes regulated mainly by the destabilisation of the corresponding transcript but not of target genes regulated by inhibition of translation. Extensive progress in protein-array technology is needed to sufficiently solve this problem. On the other hand, a lack in understanding the fundamental rules for pairing of miRNAs to their target sequences leads to deficiencies of current miRNA target prediction algorithms. This point can only be challenged by identifying and verifying several more miRNA : target gene interactions to improve the reliability of the present algorithms.

## Conclusion

Only few data about deregulated miRNA expression and its impact on tumourigenesis of malignant melanoma are available up to now. Fortunately, many groups have started to analyse miRNAs during defined processes in melanoma development and thus new data will be accessible in the near future. This kind of research will help to broaden our knowledge about melanoma development by affecting different cellular functions by targeting only one miRNA, and will also be of importance when a therapeutic approach based on miRNA is established. Obviously, there is a real chance of developing new therapeutic approaches based on miRNA biology if basic research delivers the necessary data. This is of enormous impact, especially in the case of malignant melanoma with a notably poor prognosis and a complete lack of effective standard therapies.
